# Motor Extinction: A Deficit of Attention or Intention?

**DOI:** 10.3389/fnhum.2013.00644

**Published:** 2013-10-16

**Authors:** T. David Punt, M. Jane Riddoch, Glyn W. Humphreys

**Affiliations:** ^1^School of Rehabilitation and Health Sciences, Leeds Metropolitan University, Leeds, UK; ^2^Department of Experimental Psychology, University of Oxford, Oxford, UK

**Keywords:** motor extinction, neglect, intention, attention, frontal lobe

## Abstract

Motor extinction refers to a deficit of motor production on the side opposite a brain lesion that either only becomes apparent or disproportionately worsens during bilateral motor activity. It may arise due either to a contralesional deficit in setting the motor activation level (an intentional deficit) or a deficit in contralesional awareness of the sensory consequences of movement (an attentional deficit). In this study, we investigate the nature of motor extinction in a patient (LR) with a right fronto-temporal lesion through the kinematic analysis of unimanual and bimanual circle-drawing movements. While the ipsi- and contralesional limbs performed comparably for unimanual movements, the contralesional limb demonstrated marked bradykinesia and hypometria during bimanual movements. Furthermore, these deficits were not overcome when visual feedback of the contralesional limb was provided (Experiment 1). However, when performing bimanual movements in the presence of a visual template (Experiment 2), LR was able to overcome the contralesional hypometria but not the bradykinesia which proved intractable across both experiments. Both the bradykinesia and hypometria could result from an intentional deficit of motor production. However, in Experiment 2, LR also demonstrated an abnormal level of positional drift in the contralesional limb for bimanual movements indicative of an additional attentional deficit. We conclude that LR’s presentation of motor extinction is the result of a primary intentional deficit and a secondary attentional deficit.

## Introduction

It is now generally accepted that unilateral spatial neglect (USN) involves a wide range of deficits within an overall syndrome. While the sensory and perceptual ramifications of the disorder continue to attract attention, the effects on motor control have received relatively little interest. Neglect-related movement problems take many forms but can be broadly divided into two categories; those affecting the visuo-spatial control of movement and may affect both sides of the body (see Harvey and Rossit, 2012 for a recent review), and those relating to the “underuse” of a contralesional limb. This study is concerned with the latter of these, most often referred to as “motor neglect” (Laplane and Degos, 1983; see below).

Patients who demonstrate elements of USN show a strong competitive element to their behavior that is perhaps best characterized by the related problem of “extinction,” where a contralesional stimulus fails to register awareness only when presented simultaneously with an ipsilesional stimulus (Driver and Vuilleumier, 2001). Similarly, motor extinction refers to a deficit of motor production that either worsens disproportionately or only becomes apparent when the patient is involved in bilateral activity (Punt and Riddoch, 2006; Coulthard et al., 2008). As with perceptual neglect and extinction, motor extinction is related to motor neglect, an underutilization of a limb which cannot be explained by primary motor or sensory deficits (Laplane and Degos, 1983). Motor neglect tends to be measured by clinical observation alone (Laplane and Degos, 1983; de la Sayette et al., 1989; Chamorro et al., 1997; Manabe et al., 1999) or by relatively crude clinical tests (Heilman et al., 2003). By definition, one measures motor extinction by comparing the performance of the contralesional limb on unilateral and bilateral movement tasks. Comparing performance during unilateral and bilateral movements in this way, one is able to measure the contribution of directing resources to both sides of the body even when concurrent sensory and motor deficits are present. However, the precise nature of the motor deficit may differ across cases. In some instances, contralesional hypokinesia (slowness to initiate movement) has been reported (Valenstein and Heilman, 1981; Meador et al., 1986) whereas in others contralesional impersistence (an inability to sustain a movement) has been noted (Mattingley and Driver, 1997; Mattingley, 2002). There are at least two accounts for the deficit in contralesional motor production found in motor extinction. Firstly, motor failure may be an expression of an underlying problem in monitoring the sensory consequences of movement (e.g., proprioception). For instance, it may be the case that when attentional resources are devoted to monitoring the movement of a contralesional limb alone, movements unfold in a normal manner. However, during bilateral movements, a competitive bias between the two movements may arise resulting in only ipsilesional movements being monitored effectively (proprioceptive extinction). Such an account would be in line with accounts of perceptual awareness and extinction (Driver and Vuilleumier, 2001) and would suggest an “attentional” basis for the disorder. The patient may produce equal bilateral activity but only be aware of the sensory consequences of moving the ipsilesional side. As movements unfold, the lack of awareness for contralesional movement would likely lead to a movement deficit becoming apparent.

A second possible explanation for the failure of contralesional motor activity is that it represents a failure of “intention.” Intention may be thought of as a physiological readiness to respond (Heilman et al., 2003) or the forming of a plan to move (Andersen and Buneo, 2002). Impaired intention has been linked to motor neglect, where the patient fails to automatically move the contralesional limb (Watson et al., 1978; Meador et al., 1986). In motor extinction on the other hand, intention would only fail during bilateral movement. If the underlying basis of motor extinction was isolated to one of intention, then the patient may be aware of the failure but unable to correct the problem. However, it has also been proposed that patients with a deficit in motor intention may not demonstrate normal motor awareness. Gold et al. (1994) proposed a “feed forward hypothesis” to understand anosognosia for hemiplegia, suggesting that motor intention fails in anosognosic patients. There is consequently no mismatch between the predicted and actual states of the limb as no attempt to move is made. The “forward model” of movement that this hypothesis draws on is consistent with current understanding of motor control (Wolpert et al., 1995). A recent study of patients with either anosognosia or motor neglect proposes dissociation between the two disorders with regards to the contribution of motor intention. It is suggested anosognosic patients have intact motor intention in the absence of the ability to execute movements whereas for patients with motor neglect, motor execution is spared while motor intention is impaired (Garbarini et al., 2012). Further work by the same group suggests motor awareness can be impaired in both conditions (Garbarini et al., 2013).

Of course, patients who demonstrate motor extinction may have a combination of both intentional and attentional deficits but the issue remains to be established. In this study, we examine the relation between intentional and attentional factors in motor extinction, by analyzing the performance of a patient with motor extinction on a series of unimanual and bimanual circle-drawing tasks.

### Bimanual circle-drawing movements

Circle drawing has a history of use as a method of measuring both unimanual and bimanual coordination, providing the opportunity to measure a range of parameters including amplitude, circularity, cycle duration, velocity, drift, and temporal coupling. For example, when moving bimanually, coupling is most stable when mirror-symmetrical movements are performed compared with parallel or asymmetrical movements (Semjen et al., 1995). There is also evidence that, while there is a strong tendency for synchrony, small but distinct inter-limb asynchronies arise which may be modulated by focusing visual attention toward a particular hand (Swinnen et al., 1996; Franz et al., 2002; Franz, 2004). Performance may also be affected by other factors such as hand dominance, direction of movement (Franz et al., 2002), and proprioception (Verschueren et al., 1999a).

Normal proprioception is important for optimal performance in unimanual and bimanual circle drawing. In a series of studies, Verschueren et al. (1999a,b) demonstrated the effects of proprioceptive disturbances in normal subjects on these tasks. Proprioception was disturbed by placing small vibrators (60–70 Hz) on the distal tendons of the biceps and anterior deltoid muscles while subjects performed circle drawing using the dominant limb while blindfolded. For unimanual circle drawing, tendon vibration caused the circle diameters (CDs) to be smaller; it reduced circularity and introduced a systematic drift of the hand toward the body. CDs were significantly reduced when both tendons (biceps and anterior deltoid) in the same arm were vibrated, but the reduction was relatively small (control condition = 17.63 cm, vibration of both tendons = 16.70 cm). Similar results were found for the dominant, vibrated limb when subjects performed bimanual circle drawing. Interestingly, the non-dominant, non-vibrated limb showed a significant increase in CD when the dominant limb was vibrated but again this was a relatively small change (<1 cm).

Spatial coupling is a strong feature of bimanual circle-drawing movements as demonstrated by the work of Franz (1997). Normal subjects have great difficulty in maintaining asymmetrically sized (amplitude) circles with a strong tendency for coupling. Franz argues that amplitude coupling reflects interactions at the planning (intentional) stages of movement.

Reports of the use of bimanual circle drawing to investigate bimanual coordination in subjects with brain pathology are limited, but studies relating to subjects with damage to the parietal lobe and the corpus callosum have been conducted. Serrien et al. (2001a) studied mirror or symmetrical, and parallel or asymmetrical movements in three patients with left parietal damage. The subjects showed a phase lag for the contralesional limb which was most apparent for the more difficult parallel task. Studies of subjects with acquired corpus callosum damage reveal a problem in maintaining synchronization across the limbs (Serrien et al., 2001b; Kennerley et al., 2002). Such studies add weight to the proposal that skilled bimanual coordination relies on the transmission of information from one hemisphere to the other.

In this study, we investigate the spatial and temporal characteristics of circle drawing in a subject with motor extinction. We hypothesize that contralesional unimanual movements will be relatively well-maintained. However, for bimanual movements, we predict that while ipsilesional movements will be unaffected, contralesional movements will be degraded with reduced CDs. Crucially, we measure velocity to indicate the intensity of motor production. As stated above, motor extinction may represent a contralesional deficit of proprioception (awareness) or intention under bilateral conditions, or possibly elements of both problems. Different kinematic parameters during circle drawing may be considered to primarily reflect either intentional or attentional factors. For example, movement velocity and CD can provide a measure of motor production related primarily to the intentional control of movement. Disturbing proprioception in normal subjects has only small effects on CD (see Verschueren et al., 1999b above), so that marked reductions in CD together with a reduction in movement velocity can be considered more suggestive of an intentional deficit rather than a sole deficit in awareness of the sensory consequences of movement. On the other hand, the amount of drift away from the starting position should provide a measure of the proprioceptive awareness of movement (Verschueren et al., 1999b). Drift provides a strong indication of position sense which is modulated by proprioceptive awareness.

We do not expect to find substantial difficulties with bimanual coupling but nevertheless measured the relations between temporal and spatial characteristics of the movements produced. It is important to establish whether aspects of bimanual coupling may remain even under extinction conditions. We also manipulate direction of gaze. Visual feedback will provide compensation for abnormal performance in a limb due to a deficit in proprioceptive awareness so that deficits due to poor proprioceptive awareness should decrease.

## Background

### Case study: LR

**Figure 1:**
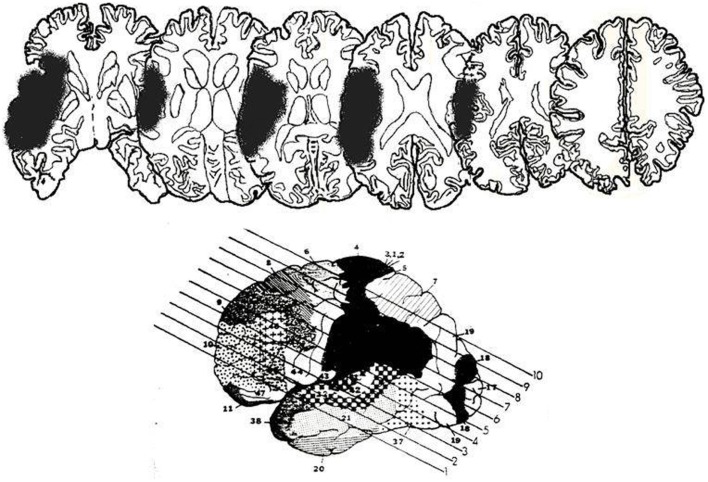
**Lesion reconstructions for LR, from MRI scan**. The lesion has been drawn onto standard slices from Gado et al. (1979). The bottom figure shows the 10 slices used. Only slices three to eight are depicted here. The left of each slice represents the right hemisphere.

LR was a previously fit 52-year-old man, formerly employed as a security guard, with a keen interest in aquarium fish and the martial arts. In June 2002, he suffered a right middle cerebral artery infarction and was hospitalized for 6 weeks. Subsequent MRI of his head showed the infarction to be primarily restricted to the right temporal lobe and posterior aspects of the right frontal lobe. More specifically, there was involvement of the inferior, middle, and superior temporal gyri on the right, and the inferior frontal and middle frontal gyri on the right (see Figure 1). LR underwent a neuropsychological screen following admission to the hospital. He also underwent additional neuropsychological testing prior to participating in the two experiments described below. Together, this information provides insights into LR’s initial difficulties and his abilities at the time of testing.

### Initial neuropsychological assessment

LR was assessed 8 days following stroke. He was oriented in time and space and performed within normal limits on picture naming, single word comprehension, complex commands, and digit span (forwards and backwards). He scored 46/54 on the Star Cancelation Test (Wilson et al., 1987), “missing” seven stars in the lower left quadrant. He scored at ceiling on tests of visual and tactile extinction. Visual extinction was tested by confrontation using the examiner’s fingers as visual stimuli either side of the examiner’s nose (central fixation). Tactile extinction was also tested by confrontation using light strokes (delivered using the examiner’s fingers) to the backs of LR’s hands (with eyes closed). LR did not present with a visual deficit.

He was tested on a novel test for motor extinction using two electronic “tappers” (WPS Electronic Tapping Test). Here, the participant places either their left, right, or both index fingers on a spring-loaded platform, and at a given signal, depresses and releases the platform as frequently as possible. The devices record the number of “taps” made in a 10-s period. When tapping with the right hand, LR made 46 taps. When tapping with the left hand, he made 41 taps. However, when tapping both hands together (each hand operating a separate device), he made 42 taps with the right hand and only 1 tap with the left hand. This pattern of performance is diagnostic of motor extinction. At this early stage post-stroke, the general impression was that LR demonstrated no language deficits, showed some deficit of executive functions as shown by impaired performance on the Brixton Test (Burgess and Shallice, 1997) and had intact memory. He showed some mild elements of neglect and in particular demonstrated motor extinction.

At this time, neurological examination revealed the following information. Muscle power was 4/5 on the left and 5/5 on the right. Assessment of tone showed no abnormalities, with equal tendon reflexes left and right. Plantar responses were downward bilaterally and there was no clonus. LR was accurate in detecting light touch and reported no differences from side to side.

### Further testing

The examination was repeated at 9 months post-stroke. At this time, the neurological examination was as above except that power appeared to have fully returned on the left (5/5). LR scored 9/9 on the Abbreviated Mental Test (Hodkinson, 1972). He performed at ceiling on tests of long term memory. Forward and backwards digit span were within normal limits. His performance on the Brixton Test for executive functions was improved but still fell within the “poor” range. LR performed normally on the Star Cancelation subtest of the Behavioral Inattention Test (Wilson et al., 1987). To assess visual attention more sensitively, LR completed a test based on the Spatial Cueing Paradigm developed by Posner et al. (1987). In this test, the subject responds to targets that can appear at locations on either side of central fixation. The appearance of a target is proceeded by a 300-ms brightening of one of these locations (50% valid and 50% invalid). In addition, targets appear at various asynchronies following the onset of the cue. Patients with lateralized attentional deficits have particular difficulties in responding to contralesional targets that follow the brightening (cueing) of the ipsilesional location. LR was slightly slower in responding to contralesional targets but the pattern for valid and invalid cues was the same on the left and right sides suggesting that he did not have a particular difficulty disengaging attention from the ipsilesional side as previously reported in patients with parietal injury and neglect (Posner et al., 1984).

LR was tested for tactile extinction using transcutaneous nerve stimulation set just above sensory threshold applied to each arm (left and right intensity thresholds were equal. Using computer-controlled presentations of these stimuli, LR was 100% accurate in responding to unilateral stimuli on the ipsilesional and contralesional sides but reported “right only” for 39% of bilateral stimuli (61% correct). He performed normally on the “sharp/dull discrimination,” “surface pressure touch,” “surface localization,” “sensory extinction,” “proprioceptive movement discrimination,” and “proprioceptive direction discrimination” subtests of the “Rivermead Assessment of Somatosensory Performance” (Winward et al., 2002).

Prior to the current experimental study, the novel tapping test for motor extinction that LR had performed during the acute phase of stroke was repeated. He now scored equal numbers of taps on the left and the right, both for unimanual and bimanual conditions (blindfolded) suggesting that he no longer demonstrated motor extinction for discrete tasks. However, as our experiments (below) demonstrate, he did continue to manifest motor extinction in continuous movement tasks (continuous circle drawing). In addition, he was also tested on the crossed-response task developed by Watson et al. (1978). This task aims to dissociate between sensory and motor neglect by demanding a response contralateral to a stimulus (e.g., the subject has to move the left arm when the right is stimulated and vice-versa). If there is no ipsilesional response to a contralesional stimulus, then the subject is considered to have a sensory deficit or sensory neglect. If there is no contralesional response to an ipsilesional stimulus, this is indicative of an exo-evoked akinesia, and suggests a motor deficit or motor neglect. LR performed at ceiling on this task.

## Experiment 1: A Comparison of Unimanual and Bimanual Circle-Drawing Movements

LR sat at a table which had no markings except for two small crosses placed 30 cm from the near edge of the table. These two crosses were equidistant from his mid-sagittal plane and were 55 cm apart. The crosses acted as start points for the circle-drawing movements to be performed. LR was instructed to draw circles rhythmically and repetitively with the extended index finger of either the left, the right, or both hands when given a start signal. Each trial lasted for 30 s and the participant was asked to maintain a constant speed and size of movement throughout the trials. In addition, there were three visual conditions where LR’s gaze position was manipulated (“look at the left hand,” “look at the right hand,” or “look at a fixation point straight ahead”). There were therefore nine different experimental conditions, and each one was performed five times (45 trials in all). The conditions were randomized across trials. All movements of the left hand were performed in an anticlockwise direction, whereas all the movements with the right hand were performed in a clockwise direction. Thus, bimanual movements were of a mirror or symmetrical type and directionally thought to relate to the natural tendencies of each hand (Franz et al., 2002). Movements were recorded using a 3-camera 3-D motion analysis system (ProReflex, Qualisys Ltd., Sweden) sampling at 200 Hz. Spherical reflective markers (5 mm diameter) were placed on the index finger nail of each hand. An auditory cue indicated the beginning and end of each trial. LR completed a small number of practice trials prior to the experimental trials in order to familiarize himself with the procedure. All trials were completed within one experimental session which lasted approximately 1 h.

### Data analysis

The *x*- and *y*-axis components of movement were analyzed offline using customized software (QTools, Qualisys Ltd., Sweden and LabVIEW, National Instruments Inc., USA). The measures of interest were the spatial and temporal characteristics of each limb and the relations between the two limbs. More specifically, we report the measurements summarized below.

#### Circle diameter

The peaks of the *x*- and *y*-axes were used to calculate CD in each plane. For the *y*-axis, each proximal peak was subtracted from the previous distal peak and for the *x-*axis, each medial peak was subtracted from the previous lateral peak.

#### Cycle duration

The mean time taken for each hand to produce a full circle was calculated.

#### Drift

Movement of the limb began with the index finger placed on the cross. As each trial progressed, any tendency for the limb to drift either in the *x*- or *y*-axis was quantified by the slope of the linear regression of displacement as a function of time.

#### Velocity

Mean velocity was calculated across each entire trial to provide a further indication of force production.

#### Inter-limb temporal coupling

The relative time that each hand reached particular landmarks was used to provide a simple indication of temporal coupling between the two limbs. The specific points used were the peaks of the *x* and *y* trajectories. The lag was calculated by subtracting the time when the right limb reached each point from the time that the left hand reached each point. Thus, a negative value refers to a “left lead” and a “right lag,” and a positive value refers to a “right lead” and a “left lag.”

## Results

For most of the analyses, mean values from each trial were treated as independent replications and submitted to a univariate analysis of variance (ANOVA). There were four factors leading to a 2 × 2 × 3 × 2 (Hand × Condition × Gaze Position × Axis) analysis. The factors were: hand (left vs. right), Condition (unimanual vs. bimanual), Gaze Position (left vs. central vs. right), and Axis (*x* vs. *y*).

### Circle diameter

**Figure 2:**
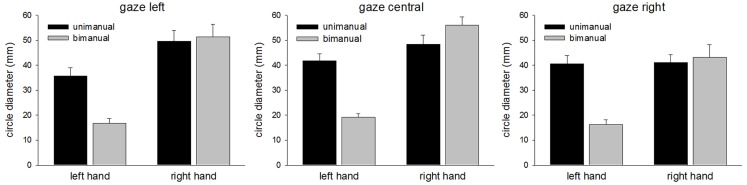
**Circle diameters for each condition in Experiment 1**.

**Figure 3:**
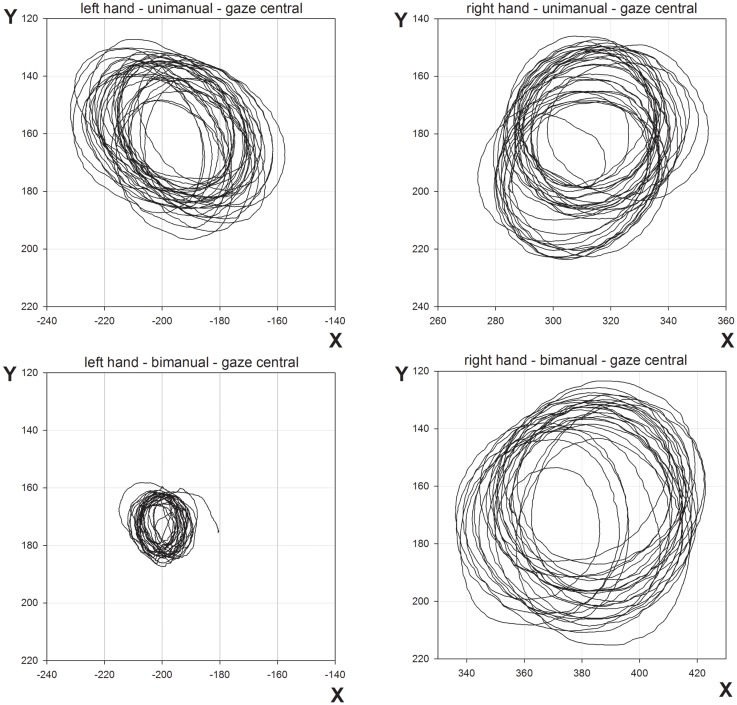
**Representative movement trajectories of unimanual and bimanual conditions from Experiment 1, when gaze was directed centrally**.

The mean CDs are shown in Figure 2. The main finding was the marked reduction in contralesional CD when LR made bimanual movements leading to a significant Hand × Condition interaction [*F*(1,96) = 37.7, *p* < 0.0001]. While unimanual CDs were within a few millimeters of each other (left = 39.3 mm, right = 46.3 mm), bimanual CDs were markedly different (left = 17.4 mm, right = 50.1 mm). There was a significant main effect of Hand [*F*(1,96) = 89.7, *p* < 0.0001] and Condition [*F*(1,96) = 18.7, *p* < 0.0001]. No other main effects or interactions proved reliable. Importantly, there was no significant main effect of Gaze Position, nor was Gaze Position involved in any significant interactions. As can be seen from Figure 2, vision failed to improve contralesional CDs when directed at the contralesional hand. CDs were comparable across all gaze position conditions. Figure 3 shows representative trajectories for unimanual and bimanual trials when vision was directed centrally.

### Cycle duration

The ANOVA revealed a significant main effect for Condition [*F*(1,48) = 4.9, *p* < 0.05]. Duration means were 891 ms for unimanual movements and 937 ms for bimanual movements. There was also a significant main effect of Gaze Position [*F*(2,48) = 7.8, *p* < 0.005] and a significant Condition × Gaze Position interaction [*F*(2,48) = 5.2, *p* < 0.01]. Further analysis showed Gaze Position was only a significant factor for the bimanual condition [*F*(2,24) = 7.7, *p* < 0.005]. Contrasts revealed cycle duration to be shorter when vision was directed to the right hand (833 ms) than when gaze position was directed centrally (986 ms) or to the left hand (993 ms) [gaze right compared with gaze central, *F*(2,24) = 11.1, *p* < 0.005; gaze right compared with gaze left, *F*(2,24) = 12.2, *p* < 0.005]. Durations were comparable when gaze was directed leftwards or centrally [*F*(2,24) < 1.0, *p* = 0.9]. For unimanual movements, there was no significant effect of Gaze Position [gaze left = 890 ms, gaze central = 904 ms, gaze right = 880 ms; *F*(2,24) < 1.0, *p* = 0.5].

### Drift

The mean slope of the linear regressions of limb position over time provided a measure of drift; the larger the number, the larger the amount of drift measured. Drift in the *x-*axis indicated movement toward or away from the mid-sagittal plane. Drift in the *y*-axis indicated movement toward or away from the body. There was a Hand × Axis interaction [*F*(1,96) = 5.8, *p* < 0.05]. Exploring the simple effects of this revealed drift in each axis to be comparable for the right hand [*x* = 0.32, *y* = 0.33, *F*(1,48) < 1.0, *p* = 0.80], whereas there was significantly more drift in the *x-*axis for the left hand [*x* = 0.62, *y* = 0.28, *F*(1,48) = 6.2, *p* < 0.025].

Directing gaze vision toward a limb reduced the amount of drift leading to a significant Hand × Gaze Position interaction [*F*(2,96) = 5.6, *p* < 0.01]. This was best explained by considering the difference in drift across the hands depending on gaze position. The left hand (0.20) drifted less than the right hand (0.37) when gaze was directed toward the left hand [*F*(1,32) = 6.8, *p* < 0.016]. When gaze was directed centrally, drift across the hands was comparable [left hand = 0.54, right hand = 0.44, *F*(1,32) < 1.0, *p* = 0.41]. The left hand (0.61) drifted more than the right hand (0.18) when gaze was directed toward the right hand [*F*(1,32) = 6.54, *p* < 0.016]. There were no other significant main effects or interactions. Importantly for this study, Condition was not found to have a significant effect on drift and neither did it appear in any interaction. While excessive drift is indicative of a proprioceptive deficit, it should be noted that in normal subjects, the non-dominant limb tends to drift more than the dominant limb and this may be sufficient to explain LR’s performance (Verschueren et al., 1999a). In comparison with the Verschueren study, LR showed increased drift in both limbs, possibly a function of the reduced circle size in this study. However, the relative drift for the dominant vs. the non-dominant hand is less in our study.

### Velocity

**Figure 4:**
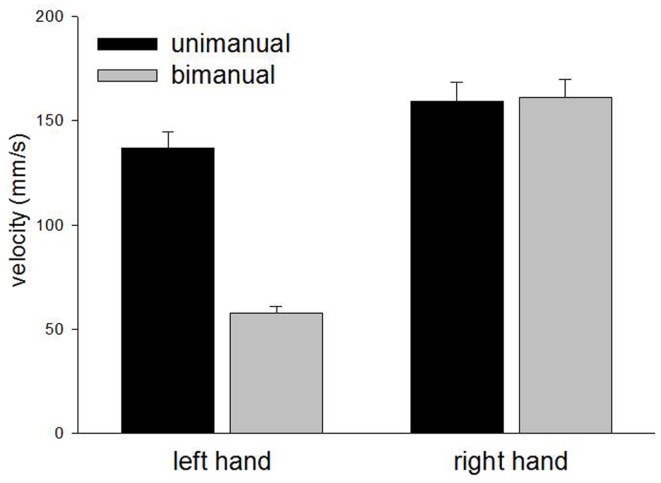
**Mean velocity for unimanual and bimanual movements in Experiment 1**.

The left and right hands demonstrated comparable velocities for unimanual movements [*F*(1,24) = 3.2, *p* = 0.09] but while the right hand maintained similar velocity for bimanual movements [*F*(1,24) < 1.0, *p* = 0.9], the left hand showed a marked reduction in velocity [*F*(1,24) = 78.8, *p* < 0.0001]. The relevant means are displayed in Figure 4. There were corresponding significant main effects of Hand [*F*(1,48) = 62.8, *p* < 0.0001], Condition [*F*(1,48) = 23.7, *p* < 0.0001], and a significant Hand × Condition interaction [*F*(1,48) = 25.9, *p* < 0.0001]. No other main effects or interactions reached significant levels.

### Inter-limb temporal coupling

Despite some of the profound asymmetries reported above, movements of the left and right hands were tightly coupled with an overall mean right lag of only 18 ms. However, there were small but significant asynchronies which were modulated by the Gaze Position [*F*(2,27) = 12.2, *p* < 0.0005]. The left hand lead was strongest when gaze was directed toward the left hand (−36 ms), less strong when gaze was directed centrally (−29 ms) and the asynchrony was reversed to a right hand lead when gaze was directed toward the right hand (12 ms). Contrasts showed a significant difference between right gaze and central gaze [*F*(1,27) = 15.3, *p* < 0.001] and between right gaze and left gaze [*F*(1,27) = 21.0, *p* < 0.0001] but not between central gaze and left gaze [*F*(1,27) < 1.0, *p* = 0.5].

## Discussion

The results from Experiment 1 show a clear deterioration in contralesional circle drawing under bimanual conditions, consistent with LR showing motor extinction. Moreover, extinction was reflected most clearly in the reduced CD and velocity, measures of motor production. This is important as it suggests that LR’s motor extinction was the result of a deficit in the intentional system that has been implicated in previous studies of motor neglect (Heilman and Valenstein, 1972; Watson and Heilman, 1979; Meador et al., 1986). We propose that an intention to move “sets” the level of activation for motor output, and LR’s clear contralesional hypometria and bradykinesia reflect difficulties in setting this level during bimanual movements. However, it may also be argued that LR showed a deficit in the awareness of movement, as CD does reduce for unimanual and bimanual circle drawing in subjects with proprioceptive disturbances (Verschueren et al., 1999a,b). Against this is the magnitude of the effects shown by LR and the normal subjects with reduced proprioception tested by Verschueren and colleagues. For example, the reduction in the proprioceptively impaired limb was <1 cm for circles drawn using a 16-cm diameter template (Verschueren et al., 1999b). Here, in Experiment 1, with no template, LRs contralesional limb reduced from 39.3 mm for unimanual movements to 17.4 mm for bimanual movements, a relatively large reduction. Also, a proprioceptive deficit would be expected to reduce accuracy in circle drawing in both directions (Meador et al., 1986) rather than the consistently hypometric movements shown by LR here. Furthermore, if a deficit in proprioceptive awareness was the primary reason for LR’s impairment, gaze position ought to have compensated in the “gaze left” condition, but this was not found. Indeed, LR was aware of the difficulties he was having with the contralesional limb when moving bimanually but was unable to correct them^1^. Such behavior is reminiscent of Meador et al.’s (1986) patient who was also described as having an intentional deficit of motor production (see General Discussion later). In addition, LR’s ipsilesional limb showed relative hypermetria in the bimanual condition (see Figures 2 and 3) possibly as a result of LR’s awareness and his attempts to correct for the hypometric movements of the contralesional limb. Further support for an intentional rather than an attentional basis for the asymmetry of bimanual movements comes from inter-limb coupling. LR generally demonstrated a “left lead” during bimanual movements which is indicative of attention being directed toward that side (Swinnen et al., 1996).

In summary, we conclude that LR does not demonstrate an attentional deficit for the sensory consequences of contralesional movements during bimanual circle drawing. Rather, his performance reflects a contralesional deficit in the maintenance of appropriate force that can generally be considered a deficit in the intentional control of movement. In LR’s case, contralesional movement initiation was preserved, but bradykinesia and hypometria became evident on bimanual movements (motor extinction).

We were surprised that LR was unable to prevent the contralesional hypometria when his vision was directed toward the left arm. To examine this further, in Experiment 2 we provided more explicit visual guidance by providing a visual template for the action (Semjen et al., 1995; Verschueren et al., 1999a; Serrien et al., 2001a; Kennerley et al., 2002). In doing this, we assessed whether, by increasing the visual cues available, we would “force” LR’s contralesional limb to make comparable movements with both limbs in the bimanual condition when gaze was directed toward the contralesional limb. Experiment 2 was performed 2 weeks after Experiment 1.

## Experiment 2: A Comparison of Unimanual and Bimanual Circle-Drawing Movements Constrained by a Visual Template

The procedure was identical to that in Experiment 1 except for the inclusion of a visual template. This template involved two circles (60 mm diameter) drawn on the table with the crosses from Experiment 1 at their center. This size of circle was chosen as it was similar to the size of the unconstrained circles performed in Experiment 1. The circles provided guidance for the movements in Experiment 2. The crosses from Experiment 1 acted as start points for each trial. The data were analyzed as for Experiment 1.

## Results

### Circle diameter

**Figure 5:**
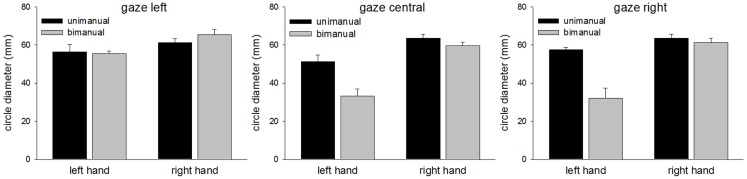
**Circle diameters for each condition in Experiment 2**.

**Figure 6:**
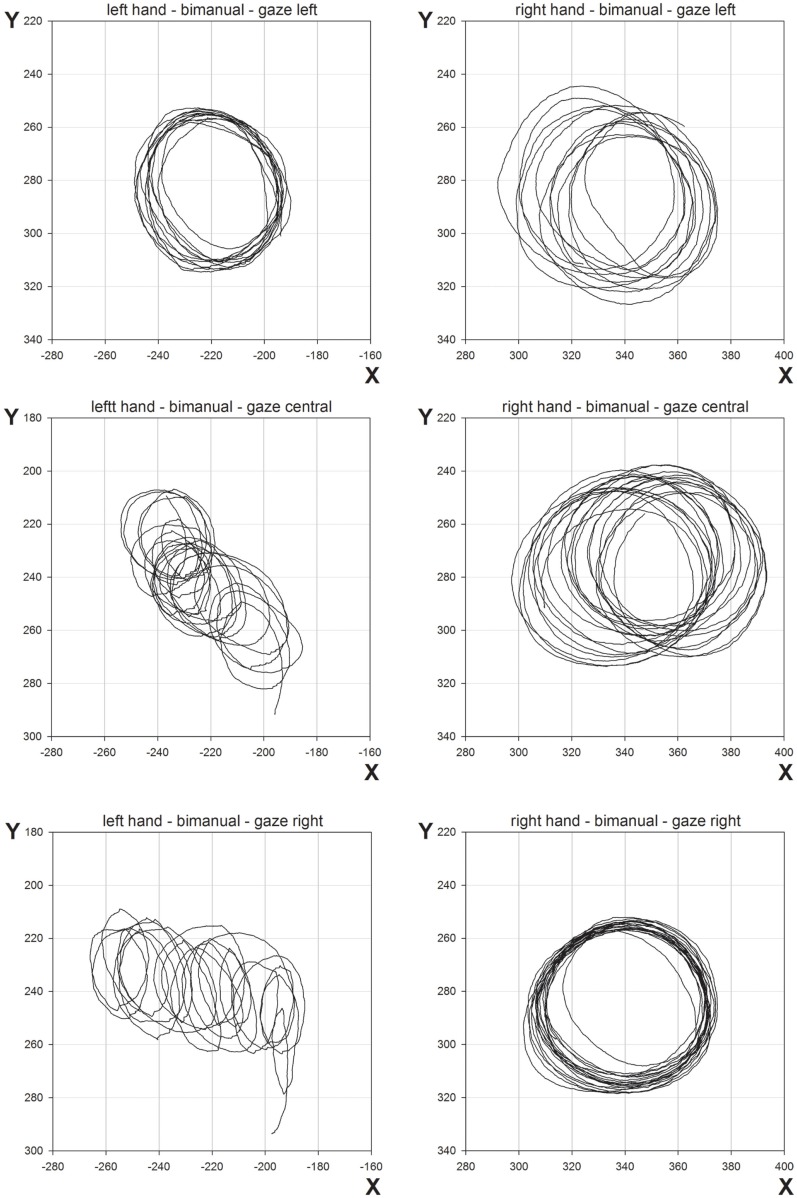
**Representative movement trajectories of bimanual conditions from Experiment 2**.

The mean CDs are shown in Figure 5. As in Experiment 1, the main finding was the marked contralesional hypometria when LR made bimanual movements. However, in Experiment 2, contralesional hypometria did not occur when gaze was directed toward the contralesional limb. Thus, provision of a visual template appeared to facilitate performance. These results are supported by significant main effects of Hand [*F*(1,96) = 77.8, *p* < 0.0001], Condition [*F*(1,96) = 21.3, *p* < 0.0001], and Gaze Position [*F*(2,96) = 7.8, *p* < 0.001]. Contrasts for Gaze Position revealed significant differences between “left gaze” and “central gaze” [*F*(1,96) = 13.9, *p* < 0.0005] and between “left gaze” and “right gaze” [*F*(1,96) = 8.8, *p* < 0.005] but not between “central gaze” and “right gaze” [*F*(1,96) < 1.0, *p* = 0.4]. Significant interactions included Hand × Condition [*F*(1,96) = 17.8, *p* < 0.0001], Hand × Gaze Position [*F*(2,96) = 4.8, *p* < 0.05], and Condition × Gaze Position [*F*(2,96) = 8.2, *p* < 0.001]. Representative bimanual trajectories from Experiment 2 are shown in Figure 6.

### Cycle duration

Cycle durations were equivalent for the left and right hands [left = 1750 ms, right = 1773 ms, *F*(1,48) < 1.0. *p* = 0.7]. Unimanual durations were shorter than bimanual durations (unimanual = 1706 ms, bimanual = 1817 ms) but this just failed to reach normal levels of significance [*F*(1,48) = 4.0, *p* = 0.05]. There was a significant main effect of Gaze Position [*F*(2,48) = 59.5, *p* < 0.0001]. Contrasts revealed that durations were on the borders of being significantly different for “central gaze” (1484 ms) and “right gaze” (1620 ms) [*F*(1,48) = 4.1, *p* = 0.05], while “left gaze” (2180 ms) was significantly different from both “central gaze” [*F*(1,48) = 105.9, *p* < 0.0001] and “right gaze” [*F*(1,48) = 68.6, *p* < 0.0001]. There were significant two-way interactions between Hand × Gaze Position [*F*(2,48) = 6.4, *p* < 0.005] and Condition × Gaze Position [*F*(2,48) = 19.0, *p* < 0.0001], and a significant three-way interaction [*F*(2,48) = 6.5, *p* < 0.005]. To understand this interaction; when gaze was directed centrally, there was no significant main effect of Hand [*F*(1,16) = 1.6, *p* = 0.2], Condition [*F*(1,16) = 1.6, *p* = 0.2], or a significant interaction [*F*(1,16) = 1.4, *p* = 0.2]. When gaze was directed rightwards, there was a clear increase in cycle duration for the right hand making unimanual movements (left = 1474 ms, right = 1957 ms), leading to a significant Hand × Condition interaction [*F*(1,16) = 18.7, *p* < 0.005]. When gaze was directed leftwards, bimanual cycle durations were clearly lengthened (left = 2471 ms, right = 2476 ms), leading to a significant main effect of Condition [*F*(1,16) = 15.8, *p* < 0.005].

### Drift

**Figure 7:**
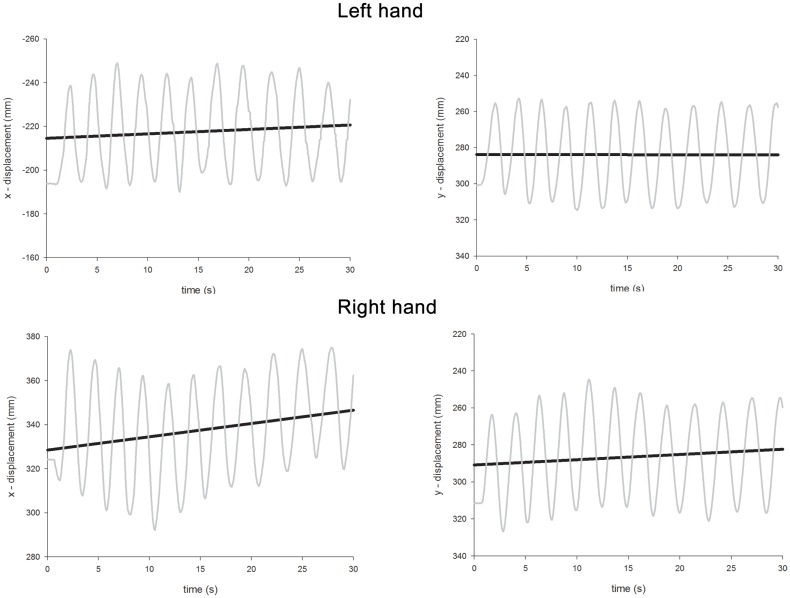
**Representative linear regression slopes for the *x*- and *y*-axes in the bimanual movement conditions when gaze was directed leftwards in Experiment 2**.

**Figure 8:**
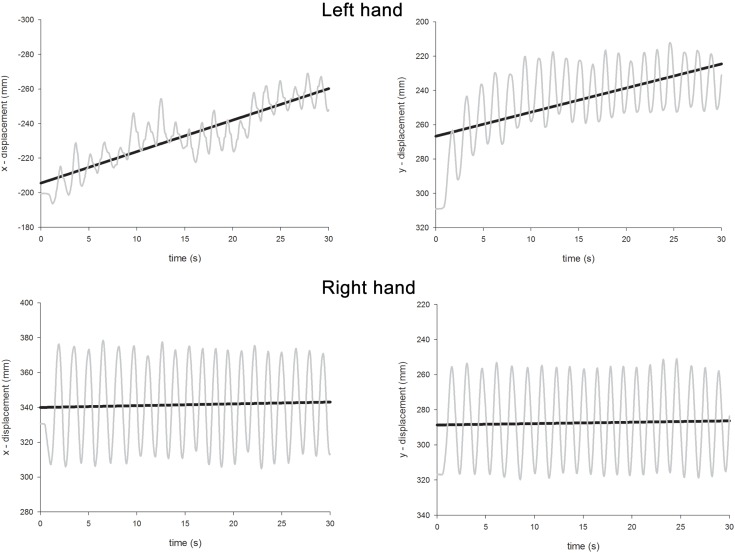
**Representative linear regression slopes for the *x*- and *y*-axes in the bimanual movement conditions when gaze was directed rightwards in Experiment 2**.

The ANOVA revealed significant main effects of Hand [the left hand drifted more than the right; left = 0.76, right = 0.34; *F*(1,96) = 40.9, *p* < 0.0001], Condition [unimanual movements drifted less than bimanual movements; unimanual = 0.38, bimanual = 0.73; *F*(1,96) = 28.6, *p* < 0.0001], and Axis [drift was more severe along the *x-*axis rather than the *y*-axis; *x*-axis = 0.67, *y*-axis = 0.44, *F*(1,96) = 12.6, *p* < 0.001]. A significant main effect of Gaze Position [*F*(2,96) = 19.4, *p* < 0.0001] was further investigated through a series of contrasts. Drift was most severe when gaze was directed centrally (0.77) and this was significantly greater than both when gaze was directed either leftwards [0.28; *F*(1,96) = 37.7, *p* < 0.0001] or rightwards [0.60; *F*(1,96) = 4.7, *p* < 0.05]. The difference between drift when gaze was directed leftwards or rightwards was also significant [*F*(1,96) = 15.8, *p* < 0.0005]. A significant Condition × Axis interaction [*F*(1,96) = 10.5, *p* < 0.005] revealed that, while drift was equivalent for each axis for unimanual movements (*x* = 0.39, *y* = 0.37), for bimanual movements, drift along the *x-*axis was much greater (*x* = 0.95, *y* = 0.51). There were also significant two-way interactions for Hand × Condition [*F*(1,96) = 21.3, *p* < 0.0001], Hand × Gaze Position [*F*(2,96) = 29.3, *p* < 0.0001], Condition × Gaze Position [*F*(2,96) = 6.3, *p* < 0.005], and a significant three-way interaction for Hand × Condition × Gaze Position [*F*(2,96) = 5.0, *p* < 0.01]. The three-way interaction occurred because contralesional drift increased disproportionately to ipsilesional drift as a function of both Condition and Gaze Position. Thus, for the right hand, drift was comparable for unimanual and bimanual movements [unimanual = 0.32, bimanual = 0.37, *F*(1,48) < 1, *p* = 0.4] and there was no Gaze Position × Condition interaction [*F*(2,48) < 1.0, *p* = 0.4]. The significant effect of Gaze Position [*F*(2,48) = 19.0, *p* < 0.0001] can be explained as follows. Visually monitoring the right limb led to reduced drift (0.11) compared with “central gaze” [0.52, *F*(1,48) = 36.1, *p* < 0.0001] and “left gaze” [0.40, *F*(1,48) = 17.6, *p* < 0.0005]. However, there was no significant difference for the “central gaze” and “left gaze” conditions [*F*(1,48) = 3.3, *p* = 0.08]. For the left hand, drift was significantly greater for bimanual movements (1.09) than unimanual movements [0.44, *F*(1,48) = 30.5, *p* < 0.0001] and here, there was a significant Condition × Gaze Position interaction [*F*(2,48) = 6.7, *p* < 0.005]. This interaction is best explained by considering the difference in drift for unimanual and bimanual movements when gaze was directed at the three possible locations. Drift was significantly greater for the left hand during bimanual movements relative to unimanual movements when gaze was directed rightwards [unimanual = 0.70, bimanual = 1.49, *F*(1,16) = 18.1, *p* < 0.005] and centrally [unimanual = 0.47, bimanual = 1.58, *F*(1,16) = 13.74, *p* < 0.005], but not when gaze was directed leftwards [unimanual = 0.13, bimanual = 0.20, *F*(1,16) = 1.74, *p* = 0.2]. Representative linear regression slopes for the *x*- and *y*-axes during bimanual movements are shown in Figure 7 (when gaze was directed leftwards) and Figure 8 (when gaze was directed rightwards).

### Velocity

As with Experiment 1, unimanual velocity was relatively equal across the hands (left = 104.85 mm/s, right = 115.98 mm/s) but there was a clear uncoupling of velocity for bimanual movements due to contralesional bradykinesia (left = 62.92 mm/s, right = 108.79 mm/s). There were associated significant main effects of Hand [*F*(1,48) = 91.8, *p* < 0.0001] and Condition [*F*(1,48) = 68.1, *p* < 0.0001], and a significant Hand × Condition interaction [*F*(1,48) = 34.1, *p* < 0.0001]. In addition, in Experiment 2, there was a main effect of Gaze Position [*F*(2,48) = 13.3, *p* < 0.0001] and a significant three-way Hand × Condition × Gaze Position interaction [*F*(2,48) = 24.4, *p* < 0.0001]. Most strikingly, while contralesional bradykinesia was found for all visual conditions when LR made bimanual movements, when directing gaze at the contralesional hand, velocity was coupled with ipsilesional velocity appearing to “follow” contralesional velocity (left = 66.21 mm/s, right = 75.82 mm/s).

### Inter-limb temporal coupling

Again, there was tight coupling of the temporal elements of bimanual circle drawing. Overall the bimanual trials, there was a right lead of just 2 ms, and asynchrony was affected by Gaze Position [*F*(2,24) = 15.5, *p* < 0.0001]. There was a left lead of 33 ms when gaze was directed centrally. This was markedly reduced to 3 ms when gaze was directed to the right [*F*(1,24) = 4.7, *p* < 0.05]. When gaze was directed to the left, there was a right lead with the left lagging behind by some 42 ms. This was significantly different to when gaze was directed to the right [*F*(1,24) = 11.4, *p* < 0.005] or centrally [*F*(1,24) = 30.6, *p* < 0.0001]. This is remarkable as one might have expected the left lead to increase with gaze toward the left rather than reverse to a left lag.

## Discussion

Experiment 2 differed from Experiment 1 in that a visual template was included. This change caused some marked differences in LR’s performance. As in Experiment 1, LR’s performance was again characterized by relatively normal unimanual movements with notable contralesional hypometria and bradykinesia on bimanual movement. However, in Experiment 2, gaze toward the contralesional hand prevented hypometria but bradykinesia persisted. Indeed, bradykinesia was shown to be the most intractable feature of bimanual contralesional performance. In addition, in Experiment 2, the contralesional limb showed a tendency to both drift as a function of both Condition and Gaze Position. That is, contralesional drift became more evident when LR moved bimanually and directed gaze away from his contralesional limb. Directing gaze at the ipsilesional right hand appeared to increase the asymmetry still further as compared with directing gaze centrally. These data suggest that, in addition to the intentional deficit apparent in the hypometric movements in Experiment 1, there was also an attentional deficit revealed. Here, visual feedback was able to compensate for the increased drift under bimanual conditions, consistent with the extra visual information compensating for reduced proprioception. In contrast, the bradykinesia remained a feature of LR’s performance. We attribute this to an intentional deficit under bimanual conditions.

Experiment 2 also showed a striking difference from Experiment 1 in terms of inter-limb coupling. While coupling was broadly similar between the two experiments when gaze was directed rightwards and centrally, there were differences when gaze was directed to the left. In Experiment 1, there was a left lead of 36 ms. In Experiment 2, this was replaced by a right lead and a left lag of 42 ms. Interestingly, this is a similar lag to that reported in three patients with left parietal damage on mirror or symmetrical circle drawing in a recent study (Serrien et al., 2001a). The study by Serrien and colleagues only addressed the temporal relationship between the limbs in a task with a visual template; the spatial relationship was not examined and the role of vision was not assessed. It seems likely that patients would direct their vision toward the “affected” limb in conditions of free vision, which would have produced a very similar situation to our condition in Experiment 2. It also further stresses the crucial role played by task constraints in temporal coupling for circle drawing (see also Franz et al., 2002).

## General Discussion

Bilateral motor function is a primary feature of human movement. This study demonstrates a patient with a right fronto-temporal lesion who was able to maintain temporal coupling but who showed a selective deficit for coupling the amplitude of movements. We interpret the deficit as a result of a competitive bias in the control of bimanual movements introduced by LR’s brain lesion – this bias reduced the intention to act with the contralesional limb when a concurrent intention to act was activated for the ipsilesional limb. The resulting bradykinesia was not influenced by visual feedback, as would be expected if it were due to reduced proprioceptive feedback. Nevertheless, LR did show evidence of “proprioceptive extinction” in Experiment 2, where there was a contralesional deficit in drift which was corrected in the presence of visual feedback (when gaze was directed to the contralesional limb). We discuss how these results relate to other patients and accounts of motor extinction.

## LR in Relation to Other Patients

LR demonstrated hypometria and bradykinesia of the contralesional limb during bimanual movements. These deficits were first described in a patient with motor neglect by Meador et al. (1986). Their patient also demonstrated a deficit in the initiation of contralesional movement (hypokinesia) not seen in LR. The patient studied by Meador et al., had suffered a hemorrhage into the right supplementary motor area (SMA) and anterior cingulate gyrus. Their explanation for the deficit was that the patient’s intentional system had been disrupted and it was argued that the right SMA may be specialized for the initiation and amplitude of movement. Motor neglect is thought to be a result of a disruption in the intentional system (Heilman, 2004) and, as with sensory neglect, has been shown to occur most frequently on the left side of the body as a result of a right-sided brain lesion (Laplane and Degos, 1983). Consistent with this, it has been shown that a lesion in the dorsolateral frontal lobe causes an intentional deficit with no related sensory deficit or sensory neglect in the crossed-response task in monkeys (Watson et al., 1978). The few reports of motor neglect and motor extinction argue for a dissociation between different motor deficits related to intention or motor planning (e.g., initiation, amplitude, velocity).

Studies relating to bilateral upper limb activity following stroke have produced conflicting results. The use of bilateral movements as a method of enhancing movement in the affected limb has become an influential approach in stroke rehabilitation (Stewart et al., 2006). However, some studies have not demonstrated such enhanced activity (Lewis and Byblow, 2004; Rice and Newell, 2004). In the case of motor extinction, by definition, affected patients will show deterioration in the performance of the affected limb. Together, these findings perhaps suggest that a “one size fits all” approach to stroke rehabilitation is inappropriate and intervention should be based on individual characteristics that patients present with.

As noted in the introduction, recent interest has centered on the motor awareness of patients with motor neglect (Garbarini et al., 2012, 2013). While we did not test this formally, it seemed clear during testing that LR was aware of the difficulties he had moving his left hand during bimanual trials. As described above, he appeared frustrated at times, occasionally “urging” his left hand to “move.” While this level of awareness has previously been reported during bimanual movements in patients with motor neglect (Meador et al., 1986; Mattingley, 2002), it stands in contrast to recent evidence suggesting a lack of motor awareness characterizes both anosognosia and motor neglect (Garbarini et al., 2013). The differing profiles of patients may reflect varying severities of motor neglect as well as varying underlying mechanisms (e.g., intention, attention). The case of LR suggests that it is possible to have a deficit in motor intention without a corresponding deficit in motor awareness.

## Motor Deficits in the Neglect Syndrome

Our study also raises issues regarding motor impairments within the neglect syndrome. Motor neglect is generally related to a deficit of intention or motor planning. However, as discussed in the introduction, deficits in either intention, attention or both may contribute to motor deficits. Just as extinction has served as a reliable measure of attentional bias in perception (Driver and Vuilleumier, 2001), so we compared unimanual vs. bimanual movements as a means of exploring similar biases in action. Our objective here was to make a first attempt in demonstrating the separation of intentional and attentional contributions to motor extinction within a single task. We hypothesized that contralesional deficits which became apparent during bimanual movements, but that could be compensated for by directing gaze toward the contralesional limb, were due to an attentional deficit. Vision would compensate for a lack of proprioceptive awareness under bimanual conditions. However, contralesional deficits during bimanual movements which were not compensated for by directing gaze toward the limb were assumed to be of intentional origin. Experiment 1 supported a purely intentional form of motor extinction. Experiment 2 also showed intention-related problems but directing gaze toward the contralesional limb led to improved performance in amplitude and drift. Only the deficit in velocity proved intractable. We interpret these results as demonstrating both intention and attention-related difficulties.

## Inter-Limb Coupling

There are at least two important issues relating to inter-limb coupling observed in LR’s performance. Firstly, one of the striking aspects of his movement was that inter-limb coupling remained ostensibly intact, despite marked asymmetries in the spatial parameters of action (e.g., amplitude). Such a dissociation is in direct contrast to callosotomy patients who can maintain spatial symmetry while temporal parameters of bimanual coordination become uncoupled (Kennerley et al., 2002). Together, these findings suggest the control of temporal and spatial elements of bimanual action are independently controlled. It was recently claimed that patients with motor neglect do not show normal spatial coupling effects when asked to simultaneously draw a line with one hand and a circle with the other (Garbarini et al., 2012); however, only movements of the ipsilesional limb were reported. Secondly, while the modulation of small asymmetries in temporal coupling as a consequence of visual guidance are generally in line with previous studies, there is one exception to this. Directing gaze toward a limb during bimanual, mirror-symmetrical movements has a tendency to either increase its lead or reduce its lag, compared with the neutral situation (Swinnen et al., 1996; Franz et al., 2002; Franz, 2004). While this was true for LR in Experiment 1 and when gaze was directed rightwards in Experiment 2, when gaze was directed leftwards in this experiment, the opposite modulation was seen with the left lag increasing. This finding is difficult to explain but suggests the correction to trajectories implemented by LR had a “knock-on” effect to temporal coupling. It is also the case, that in this particular condition, cycle duration was lengthened and this may too have had an effect on temporal coupling. The lag for the contralesional limb described above is in line with that shown by three patients with left-sided parietal lesions (Serrien et al., 2001a). However, this study neither controlled for visual guidance nor examined spatial aspects of movement. Our findings highlight the importance of vision and task constraints in bimanual circle drawing (Swinnen et al., 1996; Franz et al., 2002; Franz, 2004).

## Conclusion

In conclusion, we report the case of a patient (LR) who demonstrates motor extinction for the amplitude and velocity of movements. A comparison of unimanual and bimanual circle drawing, while manipulating gaze position, provided a means of kinematically separating movement components that reflect intentional and attentional aspects of movement. The main finding was one of contralesional bradykinesia and hypometria during bimanual activity, with the bradykinesia remaining intractable even in the presence of visual feedback. Visual feedback was able to improve secondary deficits related to attention (e.g., drift), but amplitude only normalized when direct visual guidance for action was given (i.e., a visual template). In contrast to the deficits on spatial aspects of motor performance, temporal coupling between the limbs remained. We suggest that LR demonstrates a primary deficit for intention with a secondary deficit of attention for the sensory consequences of action. Motor extinction can result from either intentional or attentional deficits in action.

## Conflict of Interest Statement

The authors declare that the research was conducted in the absence of any commercial or financial relationships that could be construed as a potential conflict of interest.

## Footnotes

^1^LR appeared frustrated during bimanual trials, complaining of the arm letting him down and occasionally telling his arm to “move.”
